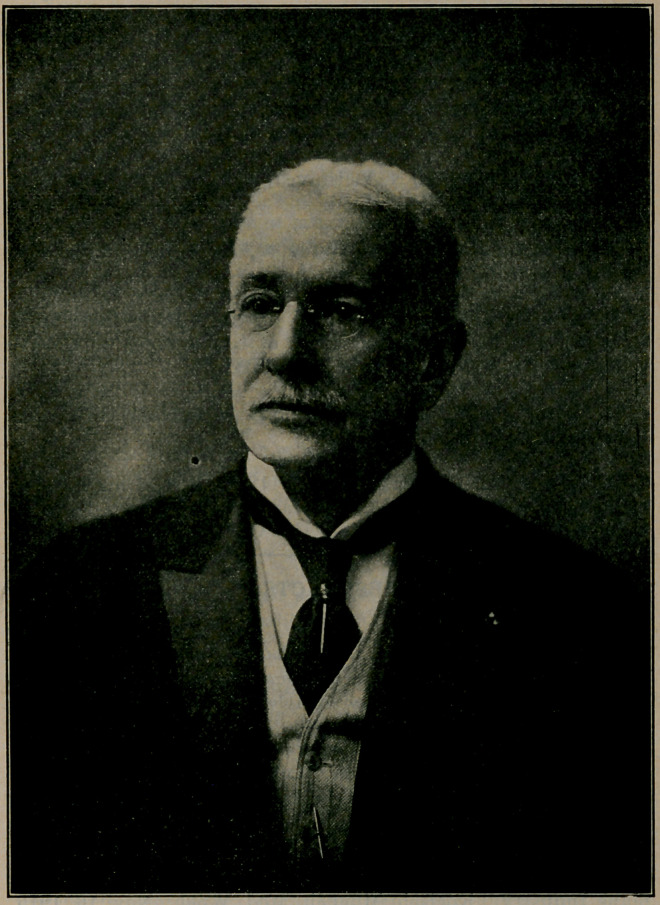# Lt.-Col. William Warren Potter, M. D.

**Published:** 1916-03

**Authors:** 


**LT.-COL. WILLIAM WARREN POTTER, M. D.,**

DIED MARCH 14, 1911, AGED 72,

Editor of this Journal from July, 1888, till his death.

## Figures and Tables

**Figure f1:**